# *Bacillus anthracis* genomic DNA enhances lethal toxin–induced cytotoxicity through TNF-α production

**DOI:** 10.1186/s12866-014-0300-9

**Published:** 2014-12-04

**Authors:** Jun Ho Jeon, Yeon Hee Kim, Min Kyung Choi, Kyung Ae Kim, Hae-Ri Lee, Jeyoun Jang, Yu-Ri Kim, Jeong-Hoon Chun, Seong Kug Eo, Tae Sung Kim, Gi-eun Rhie

**Affiliations:** Division of High-risk Pathogen Research, Center for Infectious Diseases, National Institute of Health, 187 Osongsaengmyeong 2-ro, Osong-eup, Heungdeok-gu, Cheongju-si, Chungbuk, 361-951 Republic of Korea; School of Life Sciences and Biotechnology, Korea University, Seoul, 136-701 Republic of Korea; College of Veterinary medicine and Bio-Safety Research Institute, Chonbuk National University, Jeonju, 561-765 Republic of Korea

**Keywords:** *Bacillus anthracis*, Genomic DNA, Lethal toxin, TLR9, TNF-α

## Abstract

**Background:**

*Bacillus anthracis* is the etiological agent of anthrax. Lethal toxin (LT) produced by *B. anthracis* is a well-known key virulence factor for anthrax because of its strong cytotoxic activity. However, little is known about the role of *B. anthracis* genomic DNA (BAG) in anthrax pathogenesis.

**Results:**

We examined the effect of BAG on TNF-α production and LT-mediated cytotoxicity during *B. anthracis* spore infection in mouse macrophage cell lines (RAW264.7 cells and J774A.1) and BALB/c mice. Infection of RAW264.7 cells with *B. anthracis* spores induced TNF-α expression in a multiplicity of infection (MOI)-dependent manner, and this enhancement was attenuated by the toll-like receptor (TLR) 9 inhibitor oligodeoxynucleotide (ODN)2088. BAG led to TNF-α expression in a dose- and time-dependent manner when applied to RAW264.7 cells. TNF-α expression induced by BAG was reduced by either pretreatment with TLR9 inhibitors (ODN2088 and chloroquine (CQ)) or transfection with TLR9 siRNA. Furthermore, BAG-induced TNF-α production in TLR9^+/+^ macrophages was completely abrogated in TLR9^−/−^ macrophages. BAG enhanced the phosphorylation of mitogen-activated protein kinases (MAPK), and BAG-induced TNF-α expression was attenuated by pretreatment with MAPK inhibitors. A reporter gene assay and confocal microscopy demonstrated that BAG increased NF-κB activation, which is responsible for TNF-α expression. Treatment with BAG alone showed no cytotoxic activity on the macrophage cell line J774A.1, whereas LT-mediated cytotoxicity was enhanced by treatment with BAG or TNF-α. Enhanced LT-induced lethality was also confirmed by BAG administration in mice. Furthermore, LT plus BAG-mediated lethality was significantly recovered by administration of Infliximab, an anti-TNF-α monoclonal antibody.

**Conclusions:**

Our results suggest that *B. anthracis* DNA may contribute to anthrax pathogenesis by enhancing LT activity via TLR9-mediated TNF-α production.

**Electronic supplementary material:**

The online version of this article (doi:10.1186/s12866-014-0300-9) contains supplementary material, which is available to authorized users.

## Background

*Bacillus anthracis* is a Gram-positive, spore-forming bacterium that causes anthrax [1]. Intentional use of anthrax spores as a weapon of bioterror in 2001 has provoked a need for research to find effective countermeasures [1]. When anthrax spores enter the host via diverse infection routes, macrophages, major sentinels of the immune system, serve as a reservoir of anthrax spores for germination. Macrophages then transport the bacteria to regional lymph nodes where the released bacilli multiply extensively [1,2]. Once vegetative bacilli form, they secrete high levels of exotoxins and spread systemically via the bloodstream. The exotoxins are composed of three distinct proteins, protective antigen (PA), edema factor, and lethal factor (LF), which are secreted separately as nontoxic monomers [3]. The binding of LF or edema factor to PA oligomer results in the formation of active lethal toxin (LT) or edema toxin, respectively, both of which cause massive edema, organ failure, and death of the host [1,3].

Once the pathogen invades the host, the innate immune response is the first line of defense. Innate immune cells such as macrophages and dendritic cells elicit inflammatory responses to counteract microbial infections through pattern-recognition receptors (PRR) that recognize conserved microbial structures known as pathogen-associated molecular patterns (PAMP) [4]. The best-characterized family of PRR is the TLRs, which are evolutionarily conserved from insects to humans [5]. It is well established that each TLR recognizes different sets of PAMP in bacteria or viruses. Among the PAMP that have been identified, microbial DNA shows potent immunomodulating effects on immune cells including macrophages [6]. This stimulatory effect is due to a high frequency of unmethylated CpG sequences as compared with mammalian DNA [7]. Unmethylated microbial CpG DNA from bacteria, viruses, and fungi is recognized by TLR9, which is expressed in the endosomal compartment [7]. The engagement of TLR9 subsequently recruits MyD88, which is a common adaptor molecule in TLR-mediated signaling except TLR3 [8]. The interaction of TLR9 with MyD88 in turn activates IL-1 receptor–associated kinase-4 and −1 and TNF-associated factor 6, leading to the activation of MAPKs and NF-κB to produce proinflammatory cytokines such as TNF-α and IL-6 [9].

Macrophages have been crucially implicated in *B. anthracis–*mediated pathogenesis. Once the host is infected, *B. anthracis* spores are engulfed by macrophages and induce production of TNF-α and IL-6 [10]. Some components of the vegetative form of *B. anthracis* have also been reported to be recognized by macrophages and stimulate innate immune responses [11-13]. It was previously reported that anthrolysin O, a cholesterol-dependent cytolysin in *B. anthracis,* induces TNF-α production in bone marrow–derived macrophages (BMDM) in a TLR4-dependent manner [13]. In addition, poly-γ-d-glutamic acid (PGA) capsules in *B. anthracis* elicit IL-1β production in PMA-differentiated THP-1 macrophages [11]. Furthermore, *B. anthracis* peptidoglycan (PGN), which is a major component of bacterial cell walls, stimulates TNF-α production by human monocytes [12]. Bacterial components such as PGN, LPS, and PGA sensitize LT-resistant macrophages to lethal toxin through production of TNF-α [14]. However, the role of other molecules produced by *B. anthracis* in innate immunity and pathogenesis remains unclear.

In this study, we investigated the effect of highly purified BAG on TNF-α production. Furthermore, we examined the role of BAG on LT-mediated cytotoxicity of macrophages *in vitro* and in BALB/c mice. Our results might suggest the importance of BAG in anthrax pathogenesis.

## Results

### Infection with *B. anthracis* spores enhances TLR9 mRNA and TNF-α expression

**Figure 1:**
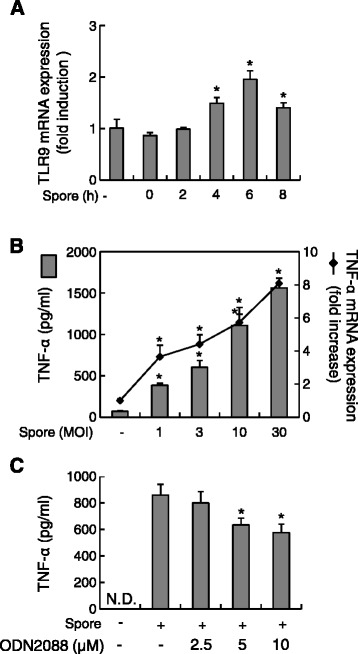
***B. anthracis*** **spore infection enhances TLR9 and TNF-α expression in RAW264.7 cells. (A)** Cells were infected with *B. anthracis* spores (MOI of 10) prepared from *B. anthracis* strain ATCC 14578 for the indicated times. At the end of the infection periods, total RNA was prepared, and TLR9 expression was analyzed using real-time RT-PCR. **(B)** RAW264.7 cells were infected with increasing numbers of *B. anthracis* spores (MOI of 1, 3, 10, or 30). TNF-α protein (bars) was analyzed by ELISA 8 h after infection; TNF-α mRNA (line) expression was analyzed by real-time RT-PCR 2 h after infection. **(C)** Cells were pretreated with ODN2088 for 1 h and then infected with *B. anthracis* spores (MOI of 10) for a further 5 h. At the end of the incubation period, culture supernatants were collected and subjected to ELISA. *P <0.05 as compared with the untreated control group (−).

To examine whether infection of macrophages with *B. anthracis* spores increases TLR9 mRNA expression, we infected RAW264.7 cells with *B. anthracis* spores (MOI of 10) for various time periods and then examined TLR9 mRNA expression using real-time RT-PCR (Figure 1A). TLR9 mRNA expression significantly increased, peaked at 6 h and decreased thereafter at 8 h (Figure 1A).

The proinflammatory cytokine TNF-α increases upon TLR9 activation [15] and plays an important role in *B. anthracis* LT-induced cytotoxicity of macrophages [14,16]. Therefore, we next investigated whether infection with *B. anthracis* spores induced the expression of TNF-α. *B. anthracis* spores significantly increased the level of both TNF-α protein and mRNA at a MOI-dependent manner (Figure 1B). Because TLR9 mRNA expression was significantly enhanced by *B. anthracis* spore infection, we examined whether TLR9 is involved in *B. anthracis* spore–induced TNF-α production. *B. anthracis* spore-induced TNF-α production was attenuated by the addition of 5 or 10 μM ODN2088, a TLR9 inhibitor, in a dose-dependent manner to 71.02% (*P* = 0.031) and 63.05% (*P* = 0.019), respectively, as compared with that of samples infected with *B. anthracis* spores only (Figure 1C). These results suggest that *B. anthracis* spores can induce TNF-α production in macrophages and that TLR9 is involved in TNF-α production following *B. anthracis* spore infection.

### BAG induces TNF-α protein and mRNA expression in a dose- and time-dependent manner

**Figure 2:**
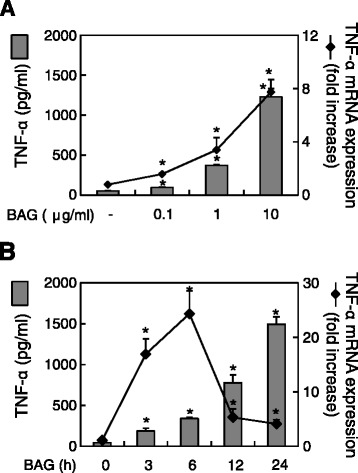
**BAG augments TNF-α protein and mRNA expression in a dose- and time-dependent manner.** RAW264.7 cells were stimulated **(A)** with various doses of BAG (0, 0.1, 1, or 10 μg/ml) for 24 h (grey bars, for protein level) or 3 h (line, for mRNA level), or **(B)** with 10 μg/ml BAG for 0, 3, 6, 12, or 24 h. At the end of the stimulation, total RNA was extracted or culture supernatants were collected. Real-time RT-PCR and ELISA were conducted to analyze BAG-induced TNF-α mRNA and protein levels, respectively. Values are the mean ± SD of three replicates per group. *P <0.05 as compared with the untreated control group.

Because *B. anthracis* spores significantly enhanced TLR9 and TNF-α expression in macrophages, we next examined whether BAG, which is known to be a TLR9 agonist [6], induced TNF-α expression at the protein and mRNA levels using ELISA and real-time RT-PCR, respectively. BAG significantly augmented the level of both TNF-α protein and mRNA at a dose-dependent fashion (Figure 2A). In time-course experiments, BAG-induced TNF-α mRNA expression increased, peaked at 6 h and declined thereafter up to 24 h. In addition, the level of TNF-α protein also increased in a time-dependent manner (Figure 2B). These results showed that BAG induced TNF-α expression at both the mRNA and protein levels.

### BAG stimulates TNF-α production in a TLR9-dependent manner

**Figure 3:**
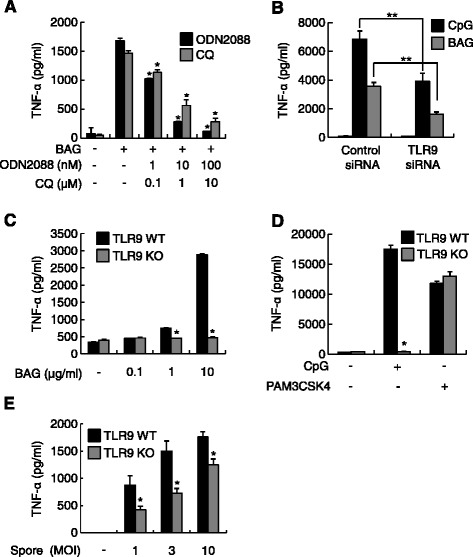
**The effect of BAG on TNF-α production is TLR9 dependent. (A)** RAW264.7 cells were pretreated with TLR9 inhibitors: 0, 1, 10, or 100 nM ODN2088 or 0, 0.1, 1, or 10 μM CQ for 1 h, followed by stimulation with 10 μg/ml BAG for an additional 24 h. **(B)** RAW264.7 cells were transfected with TLR9 siRNA or control siRNA for 48 h. Then, the cells were stimulated with 1 μM CpG ODN2395 as a positive control or 10 μg BAG for an additional 24 h. **(C)** BMDM from TLR9 WT and KO mice were stimulated with 0, 0.1, 1, or 10 μg/ml BAG for 24 h. **(D)** BMDM from TLR9 WT and KO mice were stimulated with 1 μM CpG ODN2395 or 0.1 μg/ml PAM3CSK4 for 24 h. **(E)** BMDM from TLR9 WT and KO mice were infected with increasing numbers of *B. anthracis* spores (MOI of 1, 3, or 10) for 8 h. At the end of the incubation period, culture supernatants were collected and subjected to ELISA. *P <0.05 and **P <0.01 as compared with the relevant control group.

Bacterial genomic DNA containing unmethylated CpG motifs and synthetic CpG ODN both have potent immunostimulating effects including inducing the production of proinflammatory cytokines in various cell types such as B cells, macrophages, and dendritic cells through TLR9 [17,18]. Additionally, endosomal acidification and maturation are required for full activation of TLR9 signaling pathways [7]. Therefore, we examined whether BAG-induced TNF-α production is mediated through TLR9. Both the TLR9 inhibitor ODN2088 and the endosomal acidification inhibitor CQ significantly attenuated BAG-induced TNF-α production in a dose-dependent manner (Figure 3A).

To verify that TLR9 is responsible for BAG-induced TNF-α production, we carried out TLR9 siRNA experiments. TLR9 siRNA significantly reduced the BAG- and CpG-ODN–induced TNF-α production to 45.2% (*P* = 0.0032) and 57.2% (*P* = 0.0004), respectively, as compared with results from BAG or CpG-ODN treatment in the presence of the control siRNA (Figure 3B). Additionally, to confirm the involvement of TLR9 on BAG-induced TNF-α production, we used BMDM from TLR9 WT and KO mice. BAG-induced TNF-α production was completely abolished in TLR9 KO BMDM as CpG ODN2395 but not a TLR2 ligand, PAM3CSK4 (Figure 3C, D). To further verify the role of TLR9 on *B. anthracis* spores-induced TNF-α production, we infected BMDM from TLR9 WT and KO mice with *B. anthracis* spores. Spores-induced TNF-α production from TLR9 WT BMDM was significantly attenuated in TLR9 KO BMDM (Figure 3E). The amount of TNF-α produced by spore infection in BMDM was comparable to those using J774A.1 cells (Figure 1C). These results indicate that BAG induces TNF-α production through TLR9-dependent signaling pathways.

### MAPK pathways are essential for BAG-induced TNF-α production

**Figure 4:**
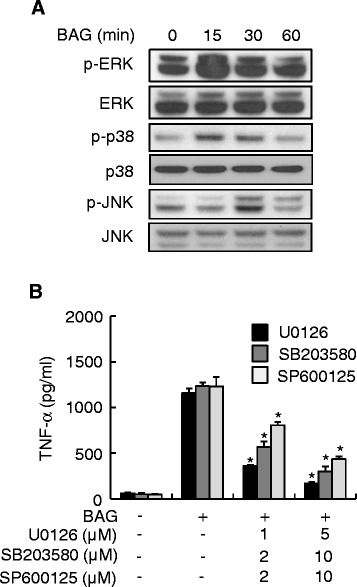
**MAPK pathways are important for BAG-induced TNF-α production. (A)** RAW264.7 cells were treated with 10 μg/ml BAG for the indicated time periods. At the end of the stimulation periods, the cells were lysed, and cell lysates were subjected to western blot analysis to determine the intracellular levels of ERK, p38, and JNK, and their phosphorylated forms. **(B)** RAW264.7 cells were pretreated with the indicated concentrations of MAPK inhibitors including ERK (U0126), p38 (SB203580), or JNK (SP600125) for 1 h, followed by stimulation with 10 μg/ml BAG for an additional 24 h. Values are the mean ± SD of triplicate samples. *P <0.05 as compared with the BAG-treated group.

Because MAPK pathways play a pivotal role in TNF-α expression [19], we examined whether BAG could induce the phosphorylation of MAPK in RAW264.7 cells using Western blotting. When the cells were stimulated with 10 μg/ml BAG for 0, 15, 30, or 60 min, phosphorylation of MAPK increased beginning at 15 min (ERK or p38) or 30 min (JNK) after BAG treatment and declined thereafter (Figure 4A). To further confirm the involvement of MAPK subtypes on BAG-induced TNF-α production, cells were pretreated with inhibitors of ERK (U0126), p38 (SB203580), or JNK kinases (SP600125). TNF-α production was attenuated by all three MAPK inhibitors in a dose-dependent manner (Figure 4B). These results indicate that MAPK pathways are critical for TNF-α production induced by BAG treatment in RAW264.7 cells.

### BAG and *B. anthracis* spores induce NF-κB activation

**Figure 5:**
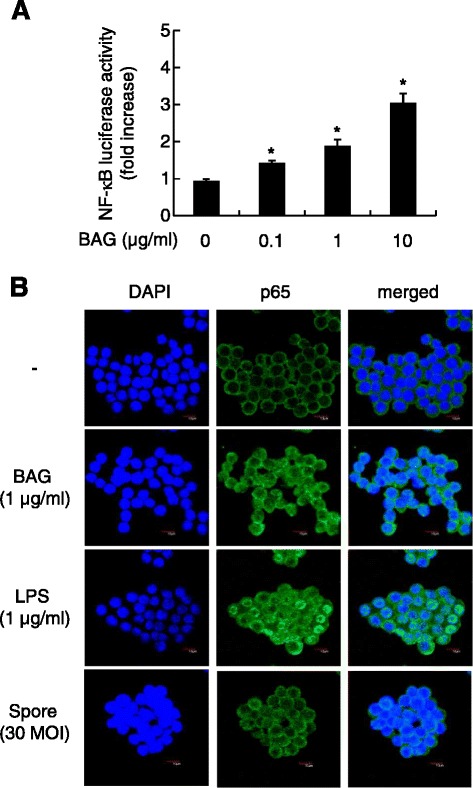
**BAG and** ***B. anthracis*** **spores induce NF-κB activation. (A)** RAW264.7 cells were cotransfected for 24 h with a firefly luciferase reporter plasmid regulated by the NF-κB transcription factor together with pRL-TK *Renilla* luciferase plasmid as an internal control for transfection efficiency. Then, the cells were stimulated with the indicated concentrations of BAG for a further 8 h. At the end of the stimulation period, the cells were lysed, and the dual luciferase activities were measured. Firefly luciferase activity was normalized to *Renilla* luciferase activity. Values are the mean ± SD of triplicate assays. *P <0.05 as compared with the untreated control group. **(B)** RAW264.7 cells were not stimulated or were stimulated with 1 μg/ml BAG or LPS or with *B. anthracis* spores (MOI of 10) for 1 h and then stained with anti-p65 followed by Alexa-488–conjugated secondary antibody and DAPI. Confocal images were obtained.

The transcription factor NF-κB is important for TNF-α gene expression [20]. Moreover, NF-κB is regulated by MAPK during TNF-α expression [21]. Thus, we investigated whether BAG could lead to NF-κB activation using an NF-κB–driven luciferase reporter system and confocal microscopic analysis of NF-κB p65 translocation to the nucleus. NF-κB luciferase activity was significantly increased by BAG stimulation in a dose-dependent manner (Figure 5A). Next, we examined whether BAG or *B. anthracis* spores could also induce NF-κB activation. Stimulation of cells with BAG or spore infection remarkably increased NF-κB activation, and this was confirmed by NF-κB p65 translocation into the nucleus as seen with anti-p65 immunostaining (Figure 5B). These results showed that BAG or spore infection induced activation of NF-κB, which is crucially involved in TNF-α gene expression.

### Treatment with BAG or TNF-α enhances the cytotoxic activity of LT on the macrophage cell line J774A.1

**Figure 6:**
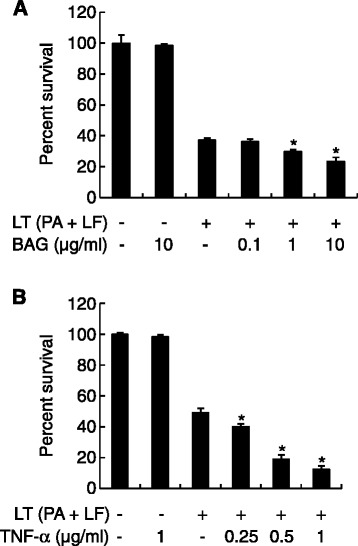
**Treatment with BAG and pretreatment with TNF-α enhance LT-mediated cytotoxicity in a concentration-dependent manner. (A)** J774A.1 cells were treated with medium alone, LT (0.5 μg/ml PA + 0.05 μg/ml LF), or LT + 0, 0.1, 1, or 10 μg/ml BAG. **(B)** J774A.1 cells were pre-treated with 0, 0.25, 0.5, or 1 μg/ml TNF-α for 24 h, and then LT (0.5 μg/ml PA +0.05 μg/ml LF) was added. After the 4-h incubation with LT, cell viability was determined using the MTT assay. The *y* axis represents the percent survival relative to control values and is given as the mean ± SD derived from three separate experiments. *P <0.05 as compared with the untreated control group.

LT is a key factor in anthrax pathogenesis, and constituents of *B. anthracis* including anthrolysin O and PGA augment the activity of LT [11,13]. Therefore, we next determined the effect of BAG on the cytotoxic activity of LT on J774A.1 cells. Treatment of cells with 10 μg/ml BAG alone did not affect the cell viability, whereas 1 or 10 μg/ml BAG with LT significantly augmented the frequency of cell death (Figure 6A). When cells were treated with 10 μg/ml BAG with LT, cell viability was significantly decreased to 63.4% (*P* = 0.0038) as compared with samples treated with LT only.

Because TNF-α enhances LT-mediated cell death [14] and because, in the current study, BAG induced substantial TNF-α secretion by macrophages, we examined whether pretreatment with TNF-α affected the cytotoxic activity of LT on J774A.1 cells. Pretreatment with 0.25, 0.5 or 1 μg/ml TNF-α significantly enhanced the cytotoxicity of LT (Figure 6B). Pretreatment with TNF-α (1 μg/ml) significantly decreased cell viability to 25.5% (*P* = 9.06 × 10^−7^) as compared with LT treatment alone. In addition, we further examined whether pretreatment of low concentrations of TNF-α can enhance the cytotoxicity of LT. Pretreatment with 7.8, 15.6, or 31.2 ng/ml TNF-α significantly enhanced the cytotoxic activity of LT, although cytotoxicity was lower than that of high concentrations of TNF-α treatment (Additional file 1: Figure S1A). Because BAG induced maximal TNF-α production in macrophages at 24 h post-stimulation, we examined whether pretreatment of BAG can also augment the cytotoxicity of LT. Pretreatment of BAG enhanced the cytotoxic activity of LT at concentrations of 1 μg/ml and 10 μg/ml (Additional file 1: Figure S1B). These results suggest that BAG plays an important role in LT*-*induced macrophage death and that TNF-α secretion induced by BAG may be involved in this cell death.

### Genomic DNA is detected during anthrax infection and enhances LT-mediated lethality in mice

**Figure 7:**
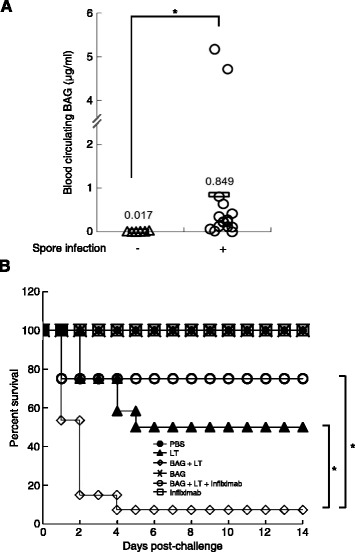
**BAG was detected during spore infection and enhanced LT-mediated lethality in mice. (A)** Six-week-old BALB/c female mice were infected with 5× LD_50_ of *B. anthracis* H9401 spores (*n* = 16) or injected with PBS (*n* = 10) into the tail vein. After 24 h, sera were isolated and filtered to remove bacilli. Genomic DNA was quantified by analyzing the signal intensity of *B. anthracis* 16S rRNA gene-specific PCR products. The relative amount was calculated based on a standard curve equation. Each triangle or circle represents a single experiment, and the horizontal bar indicates the mean. *P <0.05 as compared with the PBS-injected group. **(B)** Kaplan-Meier survival curves for BALB/c female mice injected with BAG and/or LT into the tail vein. Infliximab (1 mg) was given by intraperitoneal route before injection with LT + BAG. The percent survival was determined at 14 days after injection. Mice injected with BAG (20 μg) with LT (PA 50 μg + LF 20 μg) showed decreased survival (open diamond) when compared with LT (PA 50 μg + LF 20 μg) group (closed triangle), however, the lowered survival was recovered by injecting TNF-α inhibiting Infliximab (open circle). PBS (closed circle), BAG (20 μg, cross), and Infliximab (1 mg, open rectangle) were injected as controls. *P <0.05 as compared with the LT-BAG group.

To examine the *in vivo* relevance of BAG pathogenesis, 6-week-old BALB/c female mice were injected with 5 × LD_50_ of *B. anthracis* H9401 or PBS as a control through the tail vein. After 24 h, circulating BAG from sera was isolated, and PCR was performed using *B. anthracis* 16S rRNA gene-specific primers. In sera from 15 of 16 mice injected with *B. anthracis* spores, amplification of 16S rRNA gene-specific fragments was observed, whereas *B. anthracis* 16S rRNA was not detected in the PBS-challenged group. The amount of BAG in sera ranged from 0.027 μg/ml to 5.173 μg/ml, with an average of 0.849 μg/ml (Figure 7A).

Next, to determine the influence of BAG on LT-induced lethality *in vivo*, 6-week-old mice were given a tail vein injection of 20 μg BAG alone or with LT. Mice were then monitored for 14 days. All mice (100%; 8/8) that received PBS or 20 μg BAG alone survived, but only 50% (6/12) of the mice that received LT survived (Figure 7B). Survival rates of mice that received BAG with LT decreased significantly to 8% (1/13; *P* = 0.0016) as compared with those given LT alone (6/12). To verify whether the increase of mice lethality was due to the BAG-induced TNF-α, 1 mg of Infliximab (a TNF-α-inhibiting monoclonal antibody) was administered into each mouse before injecting BAG with LT. Consequently, the decreased survival due to the injection of BAG with LT was significantly recovered up to 75% (6/8) by injection of Infliximab (*P =* 0.0061) compared to BAG + LT group. All mice (100%; 8/8) that received Infliximab alone were alive. These results suggest that BAG produced in blood of infected mice during anthrax progress can enhance the LT-induced death by increasing TNF-α production.

## Discussion

**Figure 8:**
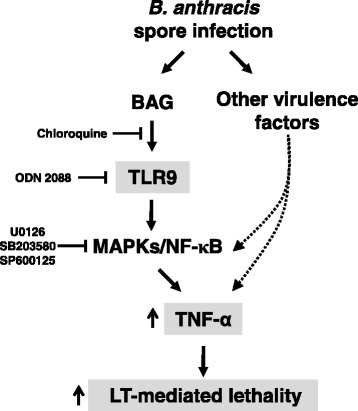
**Anthrax infection and BAG exposure enhances LT-mediated cytotoxicity in macrophages via TNF-α production and eventually increases lethality in mice.** BAG during anthrax infection is recognized by TLR9, which is inhibited by CQ or ODN2088 and induces TNF-α production via MAPK (inhibition target of U0126, SB203580, and SP600125) and NF-κB pathways. Secreted TNF-α by BAG and other virulence factors of *B. anthracis* enhances LT-mediated macrophage death and eventually increases the lethality of mice.

Innate immune responses induced by *B. anthracis* spore infection are mediated by various types of PRR including TLRs and NOD-like receptors [22-24]. In the present study, we showed that infection by fully virulent *B. anthracis* spores induced not only transcriptional upregulation of *tlr9* but also production of TNF-α in murine macrophage RAW264.7 cells. We showed that TLR9 is involved in spore-induced TNF-α production by pretreatment of RAW264.7 cells with both the endosomal acidification inhibitor CQ and the TLR9 inhibitor ODN2088. Purified BAG, a putative TLR9 ligand containing CpG motifs, led to production of TNF-α in a dose-dependent manner in both RAW264.7 cells and BMDM, and this production was mediated by MAPK and NF-кB signaling pathways via TLR9. LT-mediated macrophage cytotoxicity was enhanced by treatment with BAG and exogenous TNF-α. This effect was confirmed *in vivo* using mice. Compared with the intravenous administration of BAG that had no lethal effect, co-injection of BAG with LT increased LT-mediated lethality in mice and this effect was significantly reversed by administration of Infliximab, an anti-TNF-α monoclonal antibody. In addition, detection of BAG in the sera of anthrax spore–infected mice is consistent with a possible role for BAG-induced TNF-α in the lethality in mice. Based on these observations, we postulated that BAG and other virulence factors during anthrax infection are recognized by TLR9 and their corresponding receptors, which induce TNF-α production via MAPK and NF-кB signaling pathways. TNF-α induced by BAG and other virulence factors enhances LT-mediated cytotoxicity, which results in enhanced lethality in mice (Figure 8).

We demonstrated that DNA from *B. anthracis* elicited an inflammatory response by inducing production of the proinflammatory cytokine TNF-α in mouse macrophages. In line with our results, an immunostimulating effect of bacterial DNA has been reported, although the stimulatory potential differs among different species [25]. Genomic DNA from periodontopathogenic bacteria such as *Aggregatibacter actinomycetemcomitans* and *Porphyromonas gingivalis* stimulates macrophages to produce TNF-α and IL-6 [26]. DNA from group B streptococcus, which causes neonatal sepsis and meningitis, also activates macrophages to produce TNF-α [27]. In addition, DNA from Gram-negative *E. coli* and Gram-positive bacteria such as *Enterococcus faecalis* and *Staphylococcus aureus* triggers macrophages to release TNF-α [15]. In contrast*,* probiotic *Lactobacillus plantarum* genomic DNA does not elicit TNF-α production in the human macrophage cell line THP-1 [28]. Bacterial genomic DNA has been considered one of the principal contributors to sepsis [15]. TLR9-deficient mice are resistant to polymicrobial sepsis in an experimental peritonitis model [29]. Intravenous administration of 300 μg genomic DNA from *E. coli* to DBA/2 mice does not induce lethality, whereas 75% of mice die when given the same amount of *E. coli* DNA plus 100 μg LPS [30]. Additionally, d-galactosamine–sensitized mice, which are an *in vivo* model for sepsis, die because of macrophage-derived TNF-α, which is induced by 300 μg *E. coli* DNA or injection of 10 nmol ODN1668, a synthetic TLR9 ligand [15]. In our study, administration of 20 μg BAG alone did not alter survival rates in mice, whereas BAG with LT augmented the death of mice as compared with LT treatment alone. These results indicate that BAG can induce anthrax pathogenesis with other components such as LT, although BAG alone is not sufficient to induce lethality in mice.

LT is a major virulence factor for anthrax pathogenesis [1], and it is believed to be responsible for causing the death of infected organisms [31]. Moreover, LT induces the death of macrophages derived from certain inbred rodent strains via activation of caspase-1 and nucleotide binding domain and leucine-rich repeat containing protein 1b (NALP1b) [32]. NALP1, a member of the NOD-like receptors family, directly binds to apoptosis-associated speck-like protein containing a CARD (ASC) and caspase-1 through its pyrin domain and caspase recruitment domain, respectively, to form an inflammasome, which results in macrophage death. The *Nalp1* locus in mice possesses three paralogs, *Nalp1a*, *Nalp1b*, and *Nalp1c* [33]. *Nalp1b* is highly polymorphic in rodent strains and has been reported to determine macrophage sensitivity to LT [32]. LT rapidly induces capase-1–dependent pyroptosis in macrophages carrying the LT-sensitive allele of *Nalp1b* (*Nalp1b*^s^), whereas LT slowly induces caspase-1–independent apoptosis in LT-resistant macrophages (*Nalp1b*^R^) through cleavage of MEKs [32]. However, multiple reports have documented inconsistent LT-mediated macrophage death [14,16,31]. Indeed, LT induces apoptosis of activated macrophages with the LT-sensitive allele of *Nalp1b* (e.g., J774A.1 cells or bone marrow–derived macrophages from BALB/c mice) by inhibiting p38 kinase but not through caspase-1 activation [31]. Additionally, TNF-α produced by bacterial components promotes LT-mediated cell death in LT-resistant macrophages but not in LT-sensitive macrophages [14]. Conversely, TNF-α is involved in LT-mediated cell death in LT-sensitive bovine macrophages [16]. In agreement with this previous report, we observed greater cell death by LT and BAG than by LT alone in LT-sensitive macrophages and mice.

The inflammatory response is beneficial to the host, as it helps the host eliminate pathogens. However, excessive immune responses can be deleterious to the host because they can result in tissue damage or organ dysfunction including sepsis [34]. TNF-α is a pleiotropic cytokine that not only is involved in the growth, differentiation, and cell death of many cell types but also is implicated in pathophysiological conditions including rheumatoid arthritis, inflammatory bowel disease, atherosclerosis, and viral hepatitis [35]. TNF-α is a key mediator of septic shock syndrome and apoptosis of macrophages induced by bacterial endotoxin [36]. Treatment with TNF-α is sufficient to induce death in various cell types including hepatocytes, preadipocytes, and fibroblasts [37]. TNF-α is also crucially involved in macrophage death mediated by *B. anthracis* LT alone or LT together with PGN, PGA, or LPS [14]. Indeed, a sublytic dose of LT alone induces TNF-α secretion in macrophages, and pretreatment with a TNF-α–neutralizing antibody attenuates LT-induced macrophage death [16]. In addition, LT in combination with bacterial components such as LPS, PGN, and PGA promotes LT-mediated macrophage death, which is attenuated by pretreatment with a TNF-α–neutralizing antibody [14]. As those previous reports, administration of an anti-TNF-α monoclonal antibody, Inflximab, significantly reversed the BAG + LT-mediated lethality of mice in our study.

Recently, a number of studies have shown that LT could cause increase of vascular permeability and following vascular leakage, thereby contributing to shock and death of animals [38-41]. Bacterial constituents such as LPS and bacterial DNA have been known to induce increase of vascular permeability and leakage [42,43]. In addition, TNF-α could also induce the vascular leakage [44] and inhibitor of TNF-α reduced LPS-induced vascular leakage [43]. During anthrax infection, *B. anthracis* has been known to reach 10^7^-10^8^ organisms per milliliter of blood and induce high amount of cytokines such as TNF-α, IL-6, and IL-1β in primary macrophages and an experimental animal model [10,45]. Thus, BAG-induced TNF-α production by immune cells such as macrophages might enhance vascular leakage and septic shock, thereby contributing to lethality of an infected organism. TNF-α produced by other bacterial components described above would contribute to septic shock of an infected animal through various mechanisms.

Our results showed that BAG led to significant increase in TNF-α secretion. Therefore, TNF-α that is produced as a result of the presence of both BAG and LT may enhance LT-mediated macrophage death. In the case of *B. anthracis*, TNF-α may also be involved in spore-induced sepsis in anthrax pathogenesis. Delayed death was observed in *B. anthracis* spore–infected mice after treatment with a TNF-α antibody [46]. In our study, at 24 h post-infection with *B. anthracis* spores, the amount of BAG circulating in the blood was <0.89 μg/ml, but it may increase as anthrax infection develops in mice, resulting in TNF-α production. In our study, the maximal concentration of BAG in *in vitro* culture supernatants reached to 2.5 μg/ml when bacterial density was 8.1 × 10^5^ cfu/ml (data not shown). During anthrax infection in animal models, it was reported that the concentration of *B. anthracis* in blood may rise up to 10^7^-10^8^ cfu/ml [47]. Based on these observations including us, it is possible that the concentration of BAG in the blood of infected animal in the late phase of infection would be high enough to induce TNF-α. Additionally, it has been reported that some components of *B. anthracis* such as PGN, anthrolysin O, and PGA induced considerable amount of TNF-α in host immune cells [12,13,24]. Therefore, TNF-α secretion by BAG as well as by other components of *B. anthracis* could contribute to pathogenesis of anthrax. Therefore, we propose that the presence of BAG circulating in the blood may enhance the toxic effects of LT by increasing the TNF-α level.

## Conclusions

The current study demonstrates that BAG during anthrax infection is recognized by TLR9 and this recognition stimulates TNF-α production via MAPK and NF-κB pathways in mouse macrophages. During infection, secreted TNF-α due to BAG-mediated TLR9 activation as well as activation by other components of *B. anthracis* might enhance LT-mediated macrophage cytotoxicity and eventually increases the lethality of mice.

## Methods

### Reagents and chemicals

CpG ODN2395, TLR9 inhibitors ODN2088, and CQ were purchased from InvivoGen (San Diego, CA, USA). Recombinant TNF-α was purchased from Abcam (Cambridge, UK). All MAPK inhibitors were purchased from Calbiochem (Darmstadt, Germany). MAPK antibodies against ERK, phospho-ERK (p-ERK), p38, phospho-p38 (p-p38), JNK, and phospho-JNK (p-JNK) were from Cell Signaling Technology (Beverly, MA, USA). Dulbecco’s modified Eagle’s medium (DMEM), RPMI-1640, fetal bovine serum (FBS), and antibiotics for cell culture were purchased from Invitrogen (Carlsbad, CA, USA).

### Cell culture

The mouse macrophage cell lines RAW264.7 (TIB-71) and J774A.1 (TIB-67) were obtained from the American Type Culture Collection (ATCC; Manassas, VA, USA). The cells were cultured in DMEM supplemented with 10% FBS, 100 U/ml penicillin, and 100 μg/ml streptomycin at 37°C in a humidified incubator with 5% CO_2_. BMDMs were prepared as described previously [48]. In brief, bone marrow cells were isolated from six-week old TLR 9 wild-type (WT) (C57BL/6, Orient Bio Inc., Seoul, Korea) and TLR9 knock-out (KO) female mice which were kindly provided by professor Seong Kug Eo at Chonbuk National University by flushing marrow space of tibiae and femurs with a syringe filled with RPMI 1640. To differentiate bone marrow cells into BMDMs, the cells were then cultured in aforementioned complete DMEM with 50 μM β-mercaptoethanol in the presence of 30% L929 conditioned media as a source of M-CSF for 6–8 days at 37°C in a humidified incubator with 5% CO_2_. BMDM were trypsinized and seeded at 24 well plates for experiments.

### Spore preparation and infection

Encapsulated toxigenic *B. anthracis* ATCC 14578 (pXO1^+^ pXO2^+^) or *B. anthracis* H9401 (pXO1^+^ pXO2^+^) was streaked and incubated on blood agar plates overnight at 37°C. Spores were prepared as described [49] and stored at 4°C.

For spore infection experiments, RAW264.7 cells (1 × 10^5^ cells/ml) were seeded onto a 24-well plate in complete DMEM without antibiotics and grown overnight. The cells were washed twice with serum-free DMEM and then infected with the indicated MOI of *B. anthracis* ATCC 14578 spores for 45 min. Unphagocytosed spores were removed by washing with complete DMEM five times, and the cells were further cultured for the indicated times.

### Bacterial culture and genomic DNA preparation

*B. anthracis* ATCC 14578 genomic DNA was isolated from bacteria that were cultured on brain heart infusion plates. BAG was prepared as described [25] and stored at −20°C. To eliminate endotoxin contamination, samples were further purified using Triton X-114 as described [50]. Endotoxin levels were measured in endotoxin units per milliliter (EU/ml) using a *Limulus* amebocyte lysate assay kit (Lonza, Walkersville, MN, USA). According to this assay, 10 μg/ml DNA contained <0.1 EU/ml.

### ODN2088 and CQ treatment upon spore infection and BAG stimulation

To determine whether TLR9 is involved in TNF-α production by *B. anthracis* ATCC 14578 spores, RAW264.7 cells were pretreated with 0, 2.5, 5, or 10 μM ODN2088 for 1 h, followed by infection with *B. anthracis* spores (MOI of 10) for 5 h. Then, culture supernatants were harvested by centrifugation at 12,000 × *g* for 10 min.

To investigate whether BAG-induced TNF-α production was also mediated by TLR9, RAW264.7 cells were pretreated with 0, 1, 10, or 100 nM ODN2088 or 0, 0.1, 1, or 10 μΜ CQ, followed by stimulation with 10 μg/ml BAG for an additional 24 h. Secreted TNF-α was measured using an ELISA kit (BioLegend, San Diego, CA, USA).

### Real-time RT-PCR

RAW 264.7 cells (3 × 10^5^ cells/ml) were plated in a 6-well plate, cultured overnight, and stimulated with various concentrations of BAG for the indicated times. Total RNA was isolated from RAW264.7 cells with TRIzol reagent (Invitrogen) according to the manufacturer’s instructions. cDNA was synthesized from 5 μg total RNA using random hexamers and reverse transcriptase (Promega, Madison, WI, USA). Real-time semi-quantitative PCR was conducted using the ABI Prism® Sequence Detection System 7500 (Applied Biosystems, Foster City, CA, USA) and Power SYBR® Green PCR Master Mix (Applied Biosystems) under the following conditions: denaturation at 95°C for 1 min and amplification by cycling 40 times at 95°C for 15 s, 60°C for 15 s, and 72°C for 34 s. To determine TLR9 and TNF-α mRNA levels, the copy number for each was normalized to that of ribosomal protein L19 (L19) using the 2^−ΔΔ^*Ct* method, and then the value was compared to that of the untreated control group. The PCR primer sequences included TLR9 forward primer, 5′-ACTTCGTCCACCTGTCCAAC-3′, and reverse primer, 5′-TCATGTGGCAAGAGAAGTGC-3′; TNF-α forward primer, 5′-TCCCAGGTTCTCTTCAAGGGA-3′, and reverse primer, 5′-GGTGAGGAGCACGTAGTCGG-3′; L19 forward primer, 5′-CCAAGAAGATTGACCGCCATA-3′, and reverse primer, 5′-CAGCTTGTGGATGTGCTCCAT-3′. Real-time RT-PCR was performed in triplicate for each RNA sample. The mean ± SD of the relative TLR9 and TNF-α copy number was expressed as the fold induction and was compared with that of the untreated control group.

### Transfection with small interfering RNA (siRNA)

The siRNA that targets mouse TLR9 (ON-TARGETplus SMART-pool, L-040659-01-0005) and the non-targeting siRNA (D-001810-01-05) were purchased from Dharmacon (Lafayette, CO, USA). In brief, RAW264.7 cells (5 × 10^4^ cells/ml) were plated in a 96-well plate for 16 h and then transiently transfected with 200 nM TLR9 siRNA or control siRNA using Oligofectamine (Invitrogen) according to the manufacturer’s instructions. Four hours after transfection, the medium was replaced with complete DMEM containing 10% heat-inactivated FBS, 100 U/ml penicillin, and 100 μg/ml streptomycin. After 36 h, cells were stimulated with 10 μg/ml BAG or 1 μM CpG2395 for an additional 24 h. Then, culture supernatants were collected for TNF-α analysis with ELISA.

### Western blot analysis

RAW264.7 cells were stimulated with 10 μg/ml BAG for 0, 15, 30, or 60 min. Then, the cells were lysed with NP40 Cell Lysis Buffer (Invitrogen) on ice for 30 min. The cell lysates were collected by centrifugation at 13,000 × *g* for 10 min, and a 30 μg sample of total protein was separated by 10% SDS-PAGE and electro-transferred onto a polyvinylidene fluoride membrane. The membrane was blocked with 5% skim milk in TBS (50 mM Tris–HCl, 150 mM NaCl, pH 7.6) at room temperature for 1 h and then incubated with rabbit anti-MAPK (1:1000) at 4°C overnight. After washing three times with TBST (TBS with 0.5% Tween 20), the membrane was incubated with HRP-conjugated goat anti–rabbit IgG secondary antibody (1:3000) in blocking buffer at room temperature for 1 h. Next, the membrane was washed three times with TBST, and the immunoreactive bands were detected with ECL Western blotting reagents (Invitrogen) and X-ray film (Eastman Kodak, Rochester, NY, USA).

### TNF-α ELISA

RAW264.7 cells were plated at 5 × 10^5^ cells/ml in 96-well plates and cultured overnight in the above medium. For experiments using various inhibitors, the cells were pre-incubated with the inhibitor for 1 h, followed by stimulation with BAG for an additional 24 h. BMDM were plated at 1 × 10^6^ cells/ml in 24-well plates followed by stimulation with BAG (0, 0.1, 1, or 10 μg/ml), CpG2395 (1 μM), or PAM3CSK4 (1 μg/ml) for 24 h. At the end of the incubation period, the culture medium was collected, and the TNF-α level was analyzed using an ELISA kit (BioLegend).

### Reporter gene assay

RAW 264.7 cells (5 × 10^5^ cells/ml) were plated in 12-well plates. The cells were transfected with 1 μg pNF-κB-Luc (Clontech, Palo Alto, CA, USA) together with 0.1 μg pRL-TK *Renilla* luciferase plasmid (Promega) using LipofectAMINE and PLUS reagent (Invitrogen) according to the manufacturer’s instructions. Twenty-four hours after transfection, cells were stimulated with 0, 0.1, or 10 μg/ml BAG for a further 8 h. Cells were then lysed with reporter lysis buffer (Promega), and cell lysates were assayed for firefly and *Renilla* luciferase activity with the Dual Luciferase Reporter Assay System (Promega) in a Victor 1420 Multilabel counter (PerkinElmer Life and Analytical Sciences, Waltham, MA, USA).

### Immunofluorescence microscopy

RAW264.7 cells (1 × 10^5^/ml) were seeded on a chamber slide (Nalge Nunc, Rochester, NY, USA) and then treated with 1 μg/ml BAG and 1 μg/ml LPS for 1 h or with *B. anthracis* ATCC 14578 spores (MOI of 30) for 45 min at 37°C. After incubation for the indicated times, the cells were washed with cold PBS (GenDEPOT, Barker, TX, USA) and fixed with cold methanol for 10 min. Samples were subsequently blocked with 15% goat serum for 1 h at room temperature and washed with PBST (PBS with 0.5% Tween 20). Cells were stained with anti-NF-κB p65 primary antibody (Santa Cruz Biotechnology, Santa Cruz, CA, USA; 1:50) for 2 h at room temperature. Cells were then stained with Alexa Fluor 488–conjugated anti-mouse secondary antibody (Invitrogen; 1:100) for 1 h at room temperature and washed three times with PBST. Nuclei were stained with DAPI (Invitrogen). After being washed with PBST, samples were mounted and analyzed using an Olympus FV1000 confocal microscope (Tokyo, Japan).

### Determination of the effect of BAG and TNF-α on LT-mediated cytotoxicity

To evaluate the effect of BAG and TNF-α on LT cytotoxicity, we performed LT-mediated cytotoxicity experiments with J774A.1 cells. Monolayers of J774A.1 cells in DMEM containing 10% FBS were cultured at 37°C in 96-well plates (SPL Plastic Labware, Pochon, Korea) at 1.5 × 10^5^ cells/well. BAG was diluted with serum-free DMEM and added to the cells in the 96-well plate at final concentrations of 0 (medium alone), 0.1, 1, and 10 μg/ml. PA and LF were added to the cells at final concentrations of 0.5 μg/ml and 0.05 μg/ml, respectively. The mixtures were incubated for 1 h at 37°C before they were added to J774A.1 cells. Then, the medium was removed from the J774A.1 cell monolayers, and 100 μl of the BAG/PA/LF mixture was added to the cells for 4 h.

To examine the effect of TNF-α on LT-mediated cytotoxicity, the cells were pretreated with 0, 250, 500, or 1000 ng/ml TNF-α for 24 h, followed by treatment with PA and LF at final concentrations of 0.5 μg/ml and 0.05 μg/ml, respectively. After a 4-h incubation at 37°C in 5% CO_2_, 100 μl MTT (Sigma-Aldrich, St. Louis, MO, USA) was added to each well at a final concentration of 0.5 mg/ml. After additional incubation at 37°C for 1 h, the J774A.1 cells were lysed by adding 100 μl extraction buffer (90% isopropyl alcohol containing 25 mM HCl and 0.5% [w/v] SDS). Absorbance was then measured at 570 nm with an ELISA reader (Tecan, Männedorf, Switzerland). For each assay, controls consisted of six wells with LT only and six wells with culture medium only. Each sample was tested in duplicate and averaged for analysis.

### Quantification of BAG after infection

All animal studies were performed under the approval of the Institutional Animal Care and Use Committee of the Korea National Institute of Health. Five-week-old BALB/c female mice (Orient Bio Inc.) were housed in a specific pathogen-free environment and six-week-old mice were infected with 5× LD_50_ of *B. anthracis* H9401 spores or PBS as a control by tail vein injection. After 24 h, mice were sacrificed, and sera were isolated. For removal of bacilli, sera were filtered through 0.2-μm sterile syringe filters. Genomic DNA was isolated from sera using DNeasy Blood and Tissue Kit (QIAGEN), and PCR was performed using *B. anthracis* 16S rRNA gene–specific primers (forward, 5′-AGAGTTTGATCCTGGCTCAG-3′; reverse, 5′-AGAAAGGAGGTGATCCAGCC-3′). Serially diluted DNA (1.56 pg-5 μg) isolated from *B. anthracis* 14578 was spiked into the normal mouse blood as a standard and DNA purification procedures were performed as described above including standards. Isolated genomic DNA was used as a template, and PCR conditions were as follows: 95°C for 2 min; 30 cycles of 95°C for 30 s, 60°C for 40 s, and 72°C for 60 s; 72°C for 10 min. PCR products were separated on 1.2% agarose gels, and the signal intensity of specific bands was quantified using densitometry software (Alpha Innotech, San Jose, CA, USA). The standard curve equation was derived by analyzing signal intensities of the PCR products using known quantities of *B. anthracis* genomic DNA. Final DNA quantities were calculated by interpolating the signal intensity of the tested samples. Sequences of specific PCR-amplified bands were confirmed by sequencing.

### *In vivo* challenge with LT, BAG and TNF-α inhibitor

Five-week-old BALB/c female mice were housed in a specific pathogen-free environment (Orient Bio, Korea) and six-week-old mice were injected with LT (PA 50 μg + LF 20 μg) and/or 20 μg BAG by tail vein injection (*n* = 8-13 mice per treatment). To block the effect of BAG-induced TNF-α, Infliximab (Remicade®, Janssen Biotech Inc., Horsham, PA, USA) was used. Before injection of LT + BAG, 1 mg of Infliximab was injected into each mouse via intraperitoneal route. Mice were then monitored for survival for 14 days.

### Statistical analysis

Differences in survival between groups of mice were determined using the log-rank test with GraphPad Prism 4.0 software (GraphPad Software, Inc., San Diego, CA, USA). For other measures, the mean values ± SD were determined for each treatment group in the individual experiment. Treatment groups were compared with the appropriate control, and statistical significance was calculated with the two-tailed Student’s *t*-test. Differences were considered significant when the *P* value was <0.05.

## Additional file

Additional file 1: Figure S1. Pretreatment of low concentrations of TNF-a and BAG enhances LT-mediated cytotoxicity. (A) J774A.1 cells were pre-treated with 0, 7.8, 15.6, or 31.2 ng/ml TNF-a for 24 h, and then LT (0.1 μg/ml PA + 0.1 μg/ml LF) was added. (B) J774A. 1 cells were pre-treated with 0, 0.1, 1, or 10 μg/ml BAG for 24 h, and then LT (0.1 μg/ml PA + 0.1 μg/ml LF) was added. After the 4-h incubation with LT, cell viability was determined using MTT assay. The *y* axis represents the percent survival relative to control values and is given as the mean ± SD derived from three separate experiments. *P < 0.05 as compared with the untreated control group.

## Abbreviations

BAG: *Bacillus anthracis* genomic DNA
MOI: Multiplicity of infection
TLR: Toll-like receptor
ODN: Oligodeoxynucleotide
CQ: Chloroquine
MAPK: Mitogen-activated protein kinases
PA: Protective antigen
LF: Lethal factor
PRR: Pattern-recognition receptors
PAMP: Pathogen-associated molecular patterns
BMDM: Bone marrow-derived macrophages
PGA: Poly-γ-D-glutamic acid
PGN: Peptidoglycan
