# Supplementary material for: Bacillus anthracis genomic DNA enhances lethal toxin–induced cytotoxicity through TNF-α production
Source: BMC Microbiol. 2014 Dec 4;14:300. doi: 10.1186/s12866-014-0300-9 (PMC4267052; doi:10.1186/s12866-014-0300-9)
Supplement: Additional file 1: Figure S1. — Pretreatment of low concentrations of TNF-a and BAG enhances LT-mediated cytotoxicity. (A) J774A.1 cells were pre-treated with 0, 7.8, 15.6, or 31.2 ng/ml TNF-a for 24 h, and then LT (0.1 μg/ml PA + 0.1 μg/ml LF) was added. (B) J774A. 1 cells were pre-treated with 0, 0.1, 1, or 10 μg/ml BAG for 24 h, and then LT (0.1 μg/ml PA + 0.1 μg/ml LF) was added. After the 4-h incubation with LT, cell viability was determined using MTT assay. The y axis represents the percent survival relative to control values and is given as the mean ± SD derived from three separate experiments. *P < 0.05 as compared with the untreated control group. [file 12866_2014_300_MOESM1_ESM.pdf]

Additional file 1 : Supplementary Figure 1

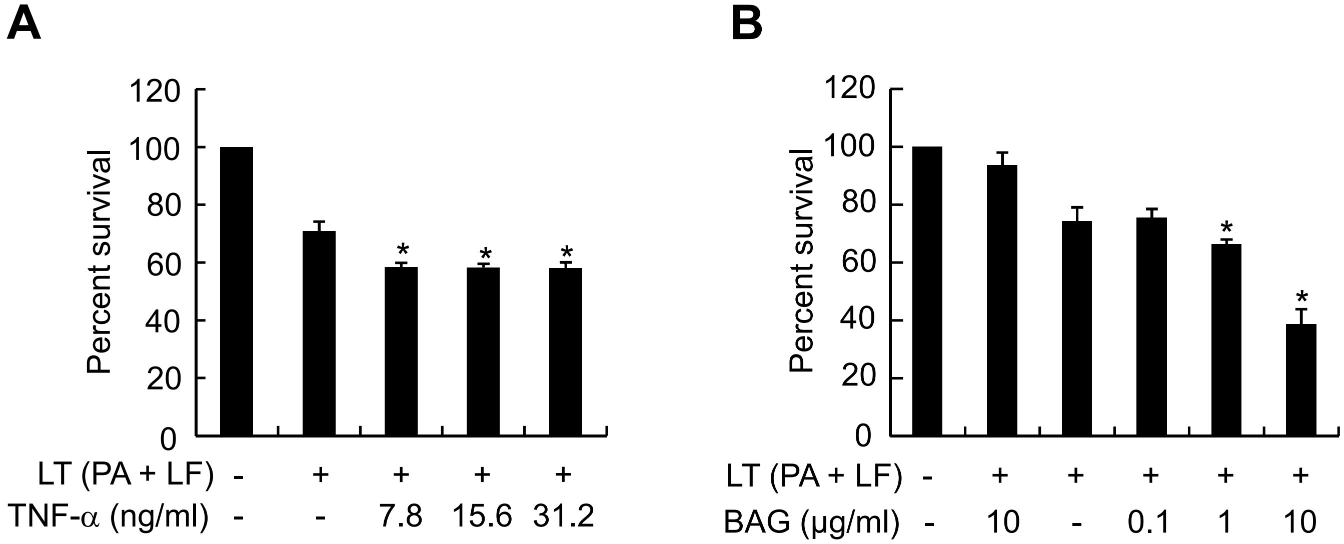

Supplementary Figure 1. Pretreatment of low concentrations of TNF-α and BAG enhances LT-mediated cytotoxicity. (A) J774A.1 cells were pre-treated with 0, 7.8, 15.6, or 31.2 ng/ml TNF-α for 24 h, and then LT (0.1 µg/ml PA + 0.1 µg/ml LF) was added. (B) J774A.1 cells were pre-treated with 0, 0.1, 1, or 10 µg/ml BAG for 24 h, and then LT (0.1 µg/ml PA + 0.1 µg/ml LF) was added. After the 4-h incubation with LT, cell viability was determined using MTT assay. The *y* axis represents the percent survival relative to control values and is given as the mean ± SD derived from three separate experiments. * $P < 0.05$ as compared with the untreated control group.
